# The bacterial cell cycle checkpoint protein Obg and its role in programmed cell death

**DOI:** 10.15698/mic2016.06.507

**Published:** 2016-03-16

**Authors:** Liselot Dewachter, Natalie Verstraeten, Maarten Fauvart, Jan Michiels

**Affiliations:** 1 Centre of Microbial and Plant Genetics, KU Leuven - University of Leuven, 3001 Leuven, Belgium.; 2 Department of Life Science Technologies, Smart Systems and Emerging Technologies Unit, imec, 3001 Leuven, Belgium.

**Keywords:** Obg, ObgE, CgtA, programmed cell death, apoptosis

## Abstract

The phenomenon of programmed cell death (PCD), in which cells initiate their own demise, is not restricted to multicellular organisms. Unicellular organisms, both eukaryotes and prokaryotes, also possess pathways that mediate PCD. We recently identified a PCD mechanism in *Escherichia coli* that is triggered by a mutant isoform of the essential GTPase ObgE (Obg of *E. coli*). Importantly, the PCD pathway mediated by mutant Obg (Obg*) differs fundamentally from other previously described bacterial PCD pathways and thus constitutes a new mode of PCD. ObgE was previously proposed to act as a cell cycle checkpoint protein able to halt cell division. The implication of ObgE in the regulation of PCD further increases the similarity between this protein and eukaryotic cell cycle regulators that are capable of doing both. Moreover, since Obg is conserved in eukaryotes, the elucidation of this cell death mechanism might contribute to the understanding of PCD in higher organisms. Additionally, if Obg*-mediated PCD is conserved among different bacterial species, it will be a prime target for the development of innovative antibacterials that artificially induce this pathway.

Although the discovery of programmed cell death (PCD) in unicellular organisms like bacteria was initially met with criticism and disbelief, the importance of bacterial PCD is increasingly being recognized by the scientific community. Many reports have demonstrated the existence of genetically encoded cell death pathways in bacteria and several different PCD mechanisms have been described. The physiological benefit of maintaining these cell death mechanisms in unicellular organism however, is still a point of speculation and ongoing debate. Many researchers believe that bacteria undergo PCD for the benefit of their kin and preservation of the bacterial population as a whole. Scientific evidence supporting this hypothesis remains limited and further research is necessary.

Serendipitous discovery of a mutant isoform of the essential GTPase ObgE led to the identification of a new bacterial PCD pathway. The gene encoding this mutant isoform carries a single point mutation, leading to the formation of ObgEK268I, further denoted as ObgE*. Interestingly – while ObgE is essential for viability – expression of ObgE* leads to a rapid and large decrease in viability. This loss of viability appears to occur through a highly coordinated process ultimately leading to cell death. During this process, an array of different PCD markers can be detected, thus classifying ObgE*-mediated cell death as a PCD mechanism. At the level of the DNA, we observed chromosome condensation and DNA fragmentation. Changes at the membrane include membrane depolarization, exposure of phosphatidylserine on the cell surface and the extensive formation of membrane blebs (shown in Figure 1).

**Figure 1:**
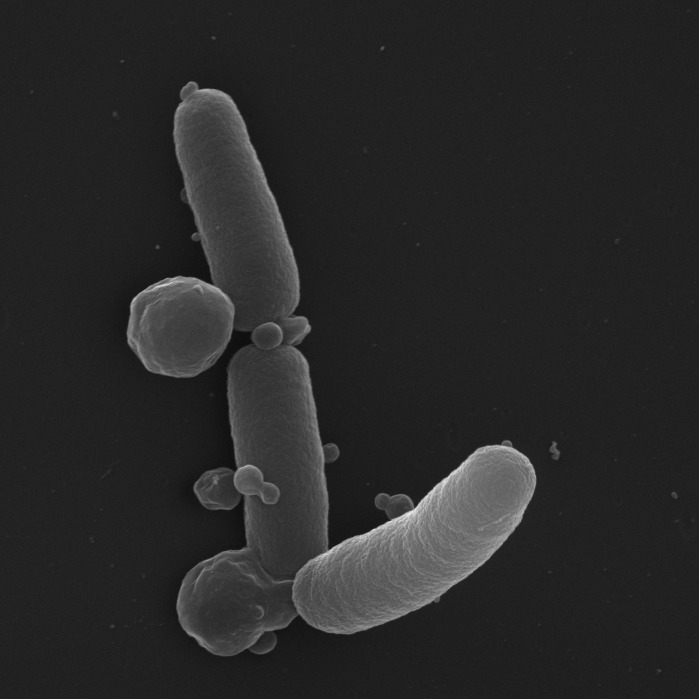
FIGURE 1: SEM image of *E. coli* undergoing ObgE*-mediated PCD. Expression of ObgE* causes the extensive formation of membrane blebs.

There is a remarkable resemblance between the physiological changes that occur during ObgE*-mediated PCD and eukaryotic apoptosis. All the PCD markers observed during the former are typical markers of apoptosis as well. Importantly, Obg is a strongly conserved protein that is also present in higher eukaryotes. Moreover, the human Obg homologue that is most similar to ObgE, ObgH1, localizes to mitochondria, the key organelles of the intrinsic pathway of apoptosis. It is therefore possible that an evolutionary link between ObgE*-mediated PCD and apoptosis exists and that our results will contribute towards a better understanding of the mechanism and evolution of PCD in eukaryotes.

The cell death mechanism triggered by ObgE* is unlike any bacterial PCD mechanism reported to date. Although it displays many physiological similarities with so called ‘apoptosis-like death’, the key regulator of the latter process, RecA, does not affect ObgE*-mediated PCD. Likewise, holin-like proteins that might facilitate autolysis are not involved in ObgE*-mediated cell death and neither is the toxin-antitoxin module *mazEF*. So far, the pathway that causes this new form of PCD remains unknown. Another unanswered research question concerns the activation of this newly discovered mechanism. In our experimental set-up we induce PCD by expression of the mutant protein ObgE*. We believe however, that this mechanism can also be triggered by wild-type ObgE under natural conditions. Possibly a post-translational modification or protein-protein interaction involving ObgE triggers cell death. Further research on ObgE*-mediated PCD is therefore also likely to contribute to our understanding of the functions of the wild-type ObgE protein.

ObgE was previously suggested to act as a cell cycle checkpoint protein. This essential GTPase is involved in ribosome assembly, the stringent response, DNA replication and chromosome segregation. It is believed that ObgE functions to link these processes and coordinate them with cell division. Since overexpression and depletion of ObgE can halt cell division, and since ObgE might also induce PCD under certain physiological conditions, it appears as though this protein acts much like eukaryotic cell cycle regulators do. These regulators are indeed also capable of inhibiting cell division or inducing PCD when the cell cycle does not proceed normally. One of the functions of these eukaryotic proteins is thus to eliminate rogue cells that could bring harm to the organism if left unchecked. What the physiological relevance and evolutionary advantage of elimination of bacterial cells with aberrant cell cycles might be, remains to be determined.

The discovery of bacterial PCD has opened up the possibility of developing a new class of antibacterials targeted at the artificial activation of PCD in bacteria. In light of the looming antibiotic crisis, such novel ways to combat bacterial infections are desperately needed. Since Obg is conserved among bacteria, the PCD mechanism triggered by ObgE* might be a prime target for the development of such novel therapeutics.

In summary, we have identified a mutant isoform of ObgE, called ObgE*, that triggers programmed cell death in *E. coli*. This PCD mechanism is distinct from other previously described bacterial PCD pathways because key regulators of these pathways were shown not to be involved in ObgE*-mediated cell death. We have therefore discovered a new mode of PCD in *E. coli*. Further investigation of this mechanism holds promise in the further understanding of the functions of wild-type ObgE, the evolution of eukaryotic PCD pathways and the development of a novel class of innovative antibacterials that exploit bacterial PCD mechanisms to combat bacterial infections. 

